# Supplementary material for: Development and validation of a framework to improve neglected tropical diseases surveillance and response at sub-national levels in Kenya
Source: PLoS Negl Trop Dis. 2021 Oct 29;15(10):e0009920. doi: 10.1371/journal.pntd.0009920 (PMC8580251; doi:10.1371/journal.pntd.0009920)
Supplement: S1 Table — (DOCX) [file pntd.0009920.s002.docx]

**S1 Table. Recommendations to improve PC-NTDs surveillance core activities**

| **THEMES** | **CODES (Recommendations)** | **Code**  **Groundedness**  **(Quotations)** |
| --- | --- | --- |
| Case detection | Provide PC-NTDs case definitions | 30 |
|  | Provide training on application of PC-NTDs case definitions | 25 |
|  | Simplified PC-NTDs case definitions | 15 |
| Case confirmation | Improved laboratory capacity | 25 |
|  |  |  |
| Reporting | Prioritising PC-NTDs reporting | 79 |
|  | Improved and updated PC-NTDs reporting tools | 69 |
|  | Enhanced training on PC-NTDs data reporting | 42 |
|  | Provision of electronic reporting tools | 36 |
|  |  |  |
| Data analysis | Enhance training on data analysis | 87 |
|  | Prioritising PC-NTDs surveillance data analysis | 24 |
|  | Frequent updates on data analysis skills | 21 |
|  | Involvement of all health cadres in surveillance activities | 17 |
|  |  |  |
| Feedback | Timely feedback on surveillance reports | 58 |
|  | Regular feedback on surveillance reports | 48 |
|  | Adopting electronic feedback mechanisms | 25 |
|  |  |  |
| Epidemic preparedness and response | Adequate outbreak response supplies | 25 |
