# Supplementary material for: Development and validation of a framework to improve neglected tropical diseases surveillance and response at sub-national levels in Kenya
Source: PLoS Negl Trop Dis. 2021 Oct 29;15(10):e0009920. doi: 10.1371/journal.pntd.0009920 (PMC8580251; doi:10.1371/journal.pntd.0009920)
Supplement: S2 Table — (DOCX) [file pntd.0009920.s003.docx]

**S2 Table. Recommendations to improve PC-NTDs surveillance support activities**

| **THEMES** | **CODES (Recommendations)** | **Code Groundedness**  **(Quotations)** |
| --- | --- | --- |
| Standards and guidelines | Availing PC-NTDs surveillance manuals | 20 |
|  | Provide guidelines for supervision | 18 |
| Supervision | Regular supervision from higher surveillance levels | 76 |
|  | Prioritising PC-NTDs supervision agenda | 59 |
|  | Training and sensitisation on surveillance supervisory activities | 29 |
|  | Provide properly constituted supervisory teams | 22 |
|  | Resource provision to facilitate supervisory activities | 22 |
|  |  |  |
| Training | Regular sensitisation of health workers on PC-NTDs surveillance | 59 |
|  | Prioritising PC-NTDs surveillance in training | 55 |
|  | Involvement of all health workers in PC-NTDs surveillance training | 31 |
|  | Providing adequate surveillance training materials | 28 |
|  | Providing frequent updates on PC-NTDs | 21 |
|  |  |  |
| Resources | Provide funding to facilitate PC-NTDs surveillance activities | 103 |
|  | Enhance human resource responsible for surveillance activities | 83 |
|  | Provision of surveillance tools and equipment | 79 |
