# Supplementary material for: Development and validation of a framework to improve neglected tropical diseases surveillance and response at sub-national levels in Kenya
Source: PLoS Negl Trop Dis. 2021 Oct 29;15(10):e0009920. doi: 10.1371/journal.pntd.0009920 (PMC8580251; doi:10.1371/journal.pntd.0009920)
Supplement: S3 Table — (DOCX) [file pntd.0009920.s004.docx]

**S3 Table. Summary of existing conceptual frameworks**

| Health Information System Frameworks | | | Public Health Surveillance System Frameworks | | |
| --- | --- | --- | --- | --- | --- |
| 1. Health Metrics Network (HMN) framework  [21] | 2. Performance of Routine Information System Management (PRISM) framework [22] | 3. Human, Organisation and Technology fit model (HOT-fit) [23] | 1. Conceptual Framework of Public Health Surveillance and Action [19] | 2. Conceptual framework to evaluate tuberculosis surveillance and action performance and measure the costs [20] | 3. Framework for evaluating public health surveillance systems for early detection of outbreaks [36] |
| -Stakeholders engagement.  -Focus on inputs, processes, outputs and outcomes of HIS. | -Assesses HIS performance considering technical, organisational and behavioral factors.  -Data management (processes), data quality and utilisation (outputs), health system performance (outcomes), and net impact on health status. | -Alignment of HIS ability, stakeholders’ practices and the organisational setting.  -Influence of information and service quality work on system use and users’ satisfaction.  -Organisational structure and environment influence system use and overall net benefits. | -Links surveillance activities to the resulting public health actions.  -Adoptable in developing settings with sub-optimally functioning disease surveillance systems. | -Disease-specific perspectives.    -Stakeholders’ inputs influence process-oriented or outcome-oriented surveillance performance indicators. | -Improved epidemic preparedness and response actions linked to processes and outputs. |
