# Supplementary material for: Development and validation of a framework to improve neglected tropical diseases surveillance and response at sub-national levels in Kenya
Source: PLoS Negl Trop Dis. 2021 Oct 29;15(10):e0009920. doi: 10.1371/journal.pntd.0009920 (PMC8580251; doi:10.1371/journal.pntd.0009920)
Supplement: S4 Table — (DOCX) [file pntd.0009920.s005.docx]

**S4 Table. Log frame 1**

| Objectives | | Indicators of achievement | Means of verification | Important assumptions |
| --- | --- | --- | --- | --- |
| Goal  (Impact) | Reduced costs for treatment interventions | Decreased number of regions and population requiring treatment | -Survey reports  -County health reports  -Community Drug Distributors (CDDs) reports | -Political goodwill  -Donor and partner support  -Community perceptions and participation |
| Purpose (Outcome) | Implementation of targeted and cost effective interventions  **(Target intervention: PC)** | -Percentage of HFs with improved reporting rates  -Percentage of HFs registering and reporting PC-NTDs cases  -Percentage of Sub-counties implementing PC-interventions informed by surveillance data | -Survey reports  -County health reports  -Record reviews  -DHIS2 | -Availability and adequacy of surveillance tools  -Submission of complete and timely reports |
| Outputs | -Accurate case registration and reporting  -Improved case confirmation capacity  -Strengthened data analysis  -Improved feedback on surveillance data  -Improved epidemic preparedness and response  -Enhance supervision on surveillance  -Improved training coverage on surveillance  -Enhanced resource capacity and support  -Improved perceptions to surveillance system  -Prioritisation of PC-NTDs surveillance activities | -Proportion of SUs registering and reporting PC-NTDs data  -Percentage of HFs with functional laboratories  -Proportion of HFs analysing PC-NTDs data  -Proportion of SUs providing feedback on PC-NTDs  -Proportion of SUs with adequate outbreak response supplies  -Proportion of SUs supervised on PC-NTDs surveillance activities  -Proportion of SUs with health workers trained on PC-NTDs surveillance  -Proportion of SU with adequate surveillance resources  -Proportion of SU with health personnel willing to be involved in PC-NTDs surveillance activities  -Proportion of SU with PC-NTDs yearly surveillance plans | -Survey reports  -County health reports  -Record reviews  -DHIS2 | -Infrastructural and technological improvements  -Availability and adequacy of surveillance tools and guidelines  -Continuous training of healthcare workers and provision of adequate training resources |
| Activities  (inputs & processes) | Technical and Organisational | -Data management (i.e. electronic reporting and data analysis tools)  -Standards and guidelines (i.e. simplified reporting guidelines, report forms, standard case definitions and data collection and analysis)  -Communication (i.e. electronic equipment and improved communication channels)  -Resource support (i.e. increased funding and provision of reporting forms) | -Survey reports  -County health reports  -County budgetary allocation reports | -Infrastructural and technological improvements  -Availability and adequacy of funding support  -Prioritising funding for PC-NTDs surveillance activities |

**DHIS2:** District Health Information System, **HFs:** Health Facilities; **SUs:** Surveillance Units (surveillance levels within sub-national structures); **PC:** Preventive Chemoprophylaxis; **PC-NTDs:** Preventive Chemotherapy-targeted Neglected Tropical Diseases
