# Supplementary material for: Development and validation of a framework to improve neglected tropical diseases surveillance and response at sub-national levels in Kenya
Source: PLoS Negl Trop Dis. 2021 Oct 29;15(10):e0009920. doi: 10.1371/journal.pntd.0009920 (PMC8580251; doi:10.1371/journal.pntd.0009920)
Supplement: S5 Table — (DOCX) [file pntd.0009920.s006.docx]

**S5 Table. Log frame 2**

| Objectives | | Indicators of achievement | Means of verification | Important assumptions |
| --- | --- | --- | --- | --- |
| Goal (Impact) | Halted disease transmission | Reduced PC-NTDs transmission hotspots | -Survey reports (i.e. TAS)  -County health reports | -Political goodwill  -County government support  -Donor and partner support  -Complete disease mapping  -Community perceptions and participation |
| Purpose (Outcome) | Enhanced identification of disease transmission hotspots  **(Target Interventions: CF)** | -Percentage of SUs registering and reporting PC-NTDs data  -Percentage of SUs notifying on PC-NTDs cases | -DHIS2  -Record reviews  -Feedback bulletins | -Availability and adequacy of surveillance tools and guidelines |
| Outputs | -Accurate case registration and reporting  -Improved case confirmation capacity  -Strengthened data analysis  -Improved feedback on surveillance data  -Improved epidemic preparedness and response  -Enhance supervision on surveillance  -Improved training coverage on surveillance  -Enhanced resource capacity and support  -Improved perceptions to surveillance system  -Prioritisation of PC-NTDs surveillance activities | Proportion of SUs registering and reporting PC-NTDs data  -Percentage of HFs with functional laboratories  -Proportion of HFs analysing PC-NTDs data  -Proportion of SUs providing feedback on PC-NTDs  -Proportion of SUs with adequate outbreak response supplies  -Proportion of SUs supervised on PC-NTDs surveillance activities  -Proportion of SUs with health workers trained on PC-NTDs surveillance  -Proportion of SUs with adequate surveillance resources  -Proportion of SUs with health personnel willing to be involved in PC-NTDs surveillance activities  -Proportion of SUs with PC-NTDs yearly surveillance plans | -Survey reports  -County health reports  -DHIS2  -Record reviews | -Infrastructural and technological improvements  -Continuous training of healthcare workers and provision of adequate training resources |
| Activities  (inputs and processes) | Human, Technical and Organisational | -Human resource management (i.e. outbreak response teams and laboratory personnel)  -Data management (i.e. provision of electronic reporting tools and listing all PC-NTDs in reporting forms)  -Standards and guidelines (i.e. updated case definitions and surveillance guidelines)  -Tools and equipment (i.e. adequately equipped laboratories)  -Resource support (i.e. increased funding support)  -Surveillance activities management (i.e. enhanced data analysis and increased community participation)  -Surveillance system attributes (i.e. considering PC-NTDs of public health importance) | -Survey reports  -County Health reports  -County Health budgetary allocation reports | -Availability and adequacy of funding support  -Prioritising funding for PC-NTDs surveillance activities |

**CF:** Case Finding; **DHIS2:** District Health Information System, **HFs:** Health Facility; **SUs:** Surveillance Units (surveillance levels within sub-national structures); **PC-NTDs:** Preventive Chemotherapy-targeted Neglected Tropical Diseases; **TAS:** Transmission Assessment Surveys
