# Supplementary material for: Development and validation of a framework to improve neglected tropical diseases surveillance and response at sub-national levels in Kenya
Source: PLoS Negl Trop Dis. 2021 Oct 29;15(10):e0009920. doi: 10.1371/journal.pntd.0009920 (PMC8580251; doi:10.1371/journal.pntd.0009920)
Supplement: S6 Table — (DOCX) [file pntd.0009920.s007.docx]

**S6 Table. Log frame 3**

| Objectives | | Indicators of achievement | Means of verification | Important assumptions |
| --- | --- | --- | --- | --- |
| Goal (Impact) | Reduced disease burden related to PC-NTDs | Decreased number of cases requiring IDM | -Survey reports  -County health reports | -Political goodwill  -County government support  -Donor and partner support  -Community perceptions and participation |
| Purpose (Outcome) | Improved estimation of overall disease burden  **(Target intervention: IDM)** | -Percentage of SUs submitting complete reports on PC-NTDs data  -Percentage of SUs with improved reporting rates | -Record reviews  -DHIS2 | -Availability and adequacy of surveillance tools and guidelines |
| Outputs | -Accurate case registration and reporting  -Strengthened data analysis  -Improved feedback on surveillance data  -Enhance supervision on surveillance  -Improved training coverage on surveillance  -Enhanced resource capacity and support  -Improved perceptions to surveillance system  -Prioritisation of PC-NTDs surveillance activities | -Proportion of HFs registering and reporting PC-NTDs data  -Proportion of HFs analysing data  -Proportion of SUs providing feedback  -Proportion of HFs supervised on NTDs  -Proportion of SUs with trained HCWs  -Proportion of SUs with adequate resources  -Proportion of SUs with health personnel willing to be involved in PC-NTD surveillance activities  -Percentage of sub-counties with PC-NTD surveillance plans | -Record reviews  -DHIS2  -Survey reports  -County health reports  -Feedback bulletins | -Complete and timely reporting  -Regular feedback and use of appropriate feedback mechanisms  -Continuous training of HCWs |
| Activities  (inputs and processes) | Human, Technical and Organisational | -Human resource management (i.e. well constituted supervisory teams)  -Data management (i.e. electronic reporting and data analysis tools)  -Standards and guidelines (i.e. updated surveillance guidelines)  -Tools and equipment (i.e. training materials and equipment and supervisory schedules)  -Communication (i.e. electronic communication channels and improved feedback mechanisms)  -Resource support (i.e. increased funding support and consistent provision of report forms)  -Strengthened management of surveillance activities (i.e. enhanced data analysis, regular feedback and supervision and increased community participation)  -Surveillance system attributes (i.e. consideration of PC-NTDs on public health importance, health management support and system adaptability) | -Survey reports  -County health reports  -County Health budgetary allocation reports | -Availability and adequacy of funding support  -Prioritising funding for PC-NTDs surveillance activities |

**DHIS2:** District Health Information System, **HCWs:** Healthcare Workers, **HFs:** Health Facilities, **IDM:** Intensified Disease Management; **SUs:** Surveillance Units (surveillance levels within sub-national structures); **PC:** Preventive Chemoprophylaxis; **PC-NTDs:** Preventive Chemotherapy-targeted Neglected Tropical Diseases
