# Supplementary material for: Development and validation of a framework to improve neglected tropical diseases surveillance and response at sub-national levels in Kenya
Source: PLoS Negl Trop Dis. 2021 Oct 29;15(10):e0009920. doi: 10.1371/journal.pntd.0009920 (PMC8580251; doi:10.1371/journal.pntd.0009920)
Supplement: Attachment — Submitted filename: Comments to the Authors-PNTD-D-21-00497.docx [file pntd.0009920.s008.docx]

This study, Arthur and colleagues: Development and validation of a framework to improve neglected tropical diseases surveillance and response at the sub-national level in Kenya. This is important study, to develop a framework to improve surveillance of NTDs. The authors have provided the details of the methods and results. They have discussed the results in a balanced way. However, I have made the following general comments to help improve the presentation of the work.

Title:

Which sub-national level? Is it County or Sub-county? Please state this level in the title and also define it clearly in the text.

Abstract:

Methods: Please state the study design applied in developing this framework.

Results: This is not a typical summary of the results from this framework, succinctly summarize the results obtained from this framework.

General comments: If you developed and validated the framework, use words that confirm the results obtained not suggestive words like “alluded to” line 48 or “appears to” line 54.

Introduction:

The introduction is long and wordy with no clear introduction of your research agenda, gaps you want addressed and how the study intend to address those gaps.

Clearly state and put to perspective any existing surveillance frameworks for PC NTDs in Kenya. State how this proposed framework will improve any of these existing frameworks.

Methods:

Phase 1: State whether the review involved engagement with the NTD Unit in Kenya to find out what surveillance framework they are using.

Phase 2: State the regions where the first study was conducted (line 229). Explain more about how primary data was collected and the methods of data collection used (how qualitative and quantitative methods were used). Please include as supplementary files, the listed study tools in line 232.

Phase 3: Explain more what Delphi survey involve and associated reference materials for this survey.

General comment: Please indicate a subheading of how data was managed and analyzed. When was this study done?

Results:

Phase 1: Summarize these findings into a flowchart/schema

Phase 4: Summarize these findings into a flowchart/schema
