# Supplementary material for: Development and validation of a framework to improve neglected tropical diseases surveillance and response at sub-national levels in Kenya
Source: PLoS Negl Trop Dis. 2021 Oct 29;15(10):e0009920. doi: 10.1371/journal.pntd.0009920 (PMC8580251; doi:10.1371/journal.pntd.0009920)
Supplement: Attachment — Submitted filename: Response to Reviewers.docx [file pntd.0009920.s009.docx]

**AUTHOR’S RESPONSE TO REVIEWERS’ COMMENTS**

**Title:** Development of a framework to improve neglected tropical diseases surveillance and response at sub-national levels in Kenya

**Authors:**

Arthur Kipkemoi Saitabau Ng'etich ([arthursaitabau@yahoo.com](mailto:arthursaitabau@yahoo.com))

Kuku Voyi ([kuku.voyi@up.ac.za](mailto:kuku.voyi@up.ac.za))

Clifford Maina Mutero ([cmutero@icipe.org](mailto:cmutero@icipe.org))

Dear Editor,

Thank you for according us an opportunity to submit a revised version of our manuscript. We appreciate the insightful feedback provided by reviewers to improve the manuscript. We have revised the manuscript accordingly considering the recommendations provided. Please find detailed below, a point-by-point response to the reviewers’ comments. The page numbers cited herein refer to the revised manuscript without track changes (clean copy).

**REVIEWER ONE**

This study, Arthur and colleagues: Development and validation of a framework to improve neglected tropical diseases surveillance and response at the sub-national level in Kenya. This is important study, to develop a framework to improve surveillance of NTDs. The authors have provided the details of the methods and results. They have discussed the results in a balanced way. However, I have made the following general comments to help improve the presentation of the work.

**Title:**

Which sub-national level? Is it County or Sub-county? Please state this level in the title and also define it clearly in the text.

**Author response:** The title has been revised to clarify the target level by using the plural form ‘sub-national levels’ that encompasses both the county and sub-county levels (Title page, page 1, line 1-3). A description of what makes up the sub-national levels has also been included within the text (Methods Section, page 9, line 221-223).

**Abstract:**

Methods: Please state the study design applied in developing this framework.

**Author response:** We utilised descriptive cross-sectional mixed-method designs to develop the framework. This has been mentioned as suggested (Abstract, page 2, line 35-36).

Results: This is not a typical summary of the results from this framework, succinctly summarize the results obtained from this framework.

**Author response:** The result section of the abstract has been revised by summarising some of the key recommendations from previous phases that informed framework components (Abstract, page 2, line 43-51).

General comments: If you developed and validated the framework, use words that confirm the results obtained not suggestive words like “alluded to” line 48 or “appears to” line 54.

**Author response:** Appropriate words have been used to confirm the study findings as suggested (Abstract, page 2, line 48 & 56).

**Introduction:**

The introduction is long and wordy with no clear introduction of your research agenda, gaps you want addressed and how the study intend to address those gaps.

**Author response:** The introduction section has been shortened and revised to clearly describe the research agenda, gaps and how the study intended to address these gaps (Introduction section, page 4-7, line 85-153).

Clearly state and put to perspective any existing surveillance frameworks for PC NTDs in Kenya. State how this proposed framework will improve any of these existing frameworks.

**Author response:** PC-NTDs surveillance within the existing IDSR system framework in Kenya has been discussed, in addition to a description of how the proposed framework will contribute towards efforts to improve the existing surveillance framework (Introduction section, page 4, line 96-100; Conclusion section, page 53, line 810-813).

**Methods:**

Phase 1: State whether the review involved engagement with the NTD Unit in Kenya to find out what surveillance framework they are using.

**Author response:** The first phase was majorly concerned with the derivation of generalised recommendations from previous surveillance assessment studies conducted in the Africa region, including initial surveillance assessments conducted in Kenya (Methods section, page 8, line 184-198). However, NTD focal persons and other concerned stakeholders at county and sub-county levels were involved in the second and third phases while stakeholders at the national NTD Unit were engaged during the framework validation phase (Page 42, line 585-596; Table 2, page 43, line 602).

Phase 2: State the regions where the first study was conducted (line 229). Explain more about how primary data was collected and the methods of data collection used (how qualitative and quantitative methods were used). Please include as supplementary files, the listed study tools in line 232.

**Author response:** The study regions, data collection methods and procedures utilised in the first study have been described as suggested (Methods section, page 9, line 214-218; page 10, line 225-230). In addition, the data collection tools have been provided as supplementary files (Methods section, page 10, line 230).

Phase 3: Explain more what Delphi survey involve and associated reference materials for this survey.

**Author response:** The Delphi survey utilised in the third phase has been described and the relevant reference materials have been cited (Methods section, page 11, line 253-256; References (Ref 33 & 34), page 58, line 961 & 964).

General comment: Please indicate a subheading of how data was managed and analyzed. When was this study done?

**Author response:** A data management and analysis section has been provided (Methods section, page 12-13, line 282-300). The study was conducted between September – December 2020.

**Results:**

Phase 1: Summarize these findings into a flowchart/schema

**Author response:** Findings for Phase 1 have been summarized using figure as suggested (Results section (Fig 2), page 14, line 320).

Phase 4: Summarize these findings into a flowchart/schema

**Author response:** Findings for Phase 4 have been summarized using table as suggested (Results section (S3 Table), page 17, line 397).

**REVIEWER TWO**

**Methods**

Is the PC-NTD framework design part of a major integrated disease surveillance and response -IDSR- program overhauling? Since the phase 1 methods are similar to those reported in reference 27.

**Author response:** Framework development was initiated by the authors based on the need to strengthen existing health information systems in line with efforts aimed at achieving the fourth strategic objective outlined in the Second National Strategic Plan for NTDs in Kenya. Reference 27 provided a detailed description of the methods adopted in the first phase of framework development, which involved a systematic review of literature on surveillance assessment studies post-adopting the revised IDSR guidelines in the African region.

The authors have as indicator of achievement of the framework Increased community participation in surveillance activities. As described, community members have been enrolled as healthcare workers for epidemiologic surveillance and they did participate in the framework´s survey phase. So, the absence of community-level representatives in the validation sessions of the framework at both Baringo and West Pokot counties (Table 2) is of some concern on ethical grounds, since they, the local communities, should be considered stakeholders in processes that affect them Can the functionaries at the validation sessions be considered community-level stakeholders?

**Author response:** Enhanced community health worker engagement with the community was considered a key facilitator of increased community participation in surveillance activities. Agreeably, local communities were considered key stakeholders in the implementation of surveillance activities at the community level. Therefore, views obtained from health workers’ working directly with the community health units were assumed to represent community needs, which are usually transmitted upwards to the sub-county level. Hence, sub-county health stakeholders’ contribution in the validation sessions were taken to be illustrative of community-level stakeholders’ opinions, given that sub-counties are decision-making points closest to the community level. However, not engaging with community level stakeholders directly in the validation phase has been acknowledged as a study limitation (Discussion section, page 52, line 795-796).

**Results**

There is an extensive step-by-step description of the framework development. Table 1 is too long, but it seems unavoidable since it shows all variables or components of the framework that were taken into account.

**Author response:** Although Table 1 (page 19, line 425) appears lengthy, it was deemed necessary to provide the reader with an in-depth description of the framework components supported by stakeholders’ perspectives (i.e. paraphrased statements).

The themes and subthemes of literature reviews in phase I are listed in detail in the text and it is tiresome. Instead, it should be presented as a table.

**Author response:** The themes and sub-themes identified in the first phase have been summarised in a figure as suggested (Results section (Fig 2), page 14, line 320).

The authors should avoid to repeat the same statement in the text and related tables as in table 2.

**Author response:** All sections that refer to Table 2 have been revised accordingly (Results section, page 41-45, line 559-629).

Figures 2, 3, 5 and 6 allow a clear understanding for the proposed framework process and interactions. But, Figure 4 is unnecessary, the information on it should be placed in the text and integrated to respective figure legends (5 and 6).

**Author response:** Figure 4 has been withdrawn from the manuscript as suggested and instead the information has been described in-text (Results section, page 40-45, line 533-629). The information has also been integrated in the figures footnotes {Fig 5 (page 45, line 631) & Fig 6 (page 46, line 633)}

**Conclusions**

The conclusion are well presented. However, it as for most of public health policies and interventions, sustainability is a key factor. The authors address that issue on the results but there is little or sparsely development in the conclusions.

**Author response:** The critical aspect of sustainability has been mentioned in the conclusion as suggested (Conclusion section, page 53, line 820-822).

**Editorial and Data Presentation Modifications**

As described in the results. Also, the author introduce some acronyms that will require a complete wording for not experts as active CF (active case finding), HIS (Hospital information system) and so.

**Author response:** The complete wording for acronyms included in the manuscript text have been provided, with all acronyms defined in full on first mention {i.e. CF (page 4, line 105; HIS (page 4, line 88)}.

**Summary and General Comments**

The authors present a comprehensive four-phase methodology for revision and proposal for development and validation of a framework for PC-NTDs surveillance and response. The manuscript presents a framework development process and a proposal that can be suitable as a blueprint for framework implementation in other countries with prevalent NTDs.

**Author response:** The reviewer’s general comment resonates with the overarching aim of the manuscript, which is to describe a framework that can be adopted at sub-national levels to improve existing surveillance and response systems not only in Kenya but also in other similar NTD-endemic settings.

**REVIEWER THREE**

**Methods**

The methods section is detailed and well aligned to the goal of the study. It is adequate especially regarding the objectives of the study. It however, gets repeated almost totally in the results section and so a way of preventing this should have been devised in order to prevent this repetition.

**Author response:** The results section has been revised as suggested to avoid repetition of information already mentioned in the preceding methods section (Results section, page 13-17, line 301-402).

**Results**

The analysis as presented does match the analysis plan and clearly answers the research question according to the plan provided. The results though clear are too detailed, long and repetitive of earlier text in the manuscript. A means of summary the results to provide more focus and make it easier to read and follow should be explored.

**Author response:** The results section has been reworked for clarity by focusing on the main findings from the preceding study phases that informed development of the framework (Results section, Page 13-46, line 301-633).

The tables are of sufficient quality though they also provide too much information and details instead of being a summary of the findings. I would also suggest that the writers consider moving some of the less relevant tables to the appendix.

**Author response:** As suggested, some tables have been withdrawn from the manuscript and instead included as supplementary material {S1 Table & S2 Table (page 14, line 330); S3 Table (page 17, line 397); S4 Table, S5 Table & S6 Table (page 42, line 590)}. However, Table 1 & 2 have been retained in the manuscript since they provide vital details that informed the frameworks’ components aided by participants’ paraphrased statements (page 19, line 425 & page 43, line 602 respectively).

**Conclusions**

The conclusions are supported by the data and unlike the methods and results sections the conclusions are more concise. The limitations of the study were discussed as well and the authors clearly indicated how the findings of the study would be used. However, the utility of the finding to WHO who provide technical guidance to the country programmes is not articulated in the paper.

**Author response:** Additional text has been provided in the conclusion describing how WHO Regional Office for Africa and national health ministries could utilise findings from the study (Conclusion section, page 54, line 829-832).

**Editorial and Data Presentation Modifications**

The manuscript is too long, a bit repetitive in presentation, and difficult to read. The introduction and the methods section are very detailed and most of the information and data under methods gets repeated in the results section instead of presenting a summary in the methods. The manuscript could benefit from.

**Author response:** All relevant sections of the manuscript (i.e. introduction, methods and results) have been reworked as suggested.

**Summary and General Comments**

The concept is perfect and focused on providing an M&E framework for NTDs which is a laudable goal and missing on many NTD programmes. The manuscript demonstrates a good understanding of M&E broadly and how it can be applied for NTDs M&E for best results and decision-making. Generally, the paper is highly informative, and the writers have done a great job but as indicated it is too long and makes it difficult to read and follow. I would suggest that the manuscript is broken up into more than one paper. Many of the tables could also be put in the appendix. I suggest a major review of the paper before considering it for publication despite its utility.

**Author response:** The authors initially thought of splitting the manuscript into two papers, with the first paper intended to describe the proposed framework while the second was to focus on validation of the framework. However, to make the framework development process comprehensible and easy for the reader to follow, the authors opted to describe the full process in a single paper. Therefore, in trying to make the paper easy to read, various sections of the paper have been shortened and some of the tables have been moved to appendices (i.e. included as supplementary material) as suggested.

Under the results section, the attributes of the surveillance functions need to be appropriately defined since they overlap in definition noting that the WHO, the organization which is responsible for providing technical norms and guidance has specific definitions for these attributes. An example is the attributes of data quality which includes accuracy, completeness, reliability, and timeliness and many of the other attributes are covered in meaning and definition under these attributes of quality data. The paper should therefore be able to define on its own the different expected data attributes to serve as guidance for the reader and at least should be provided in the introduction or literature search section.

**Author response:** Clear definitions for surveillance attribute functions assessed in the paper have been provided in reference to definitions outlined in WHO and CDC guidelines (Methods section, page 10, line 233-242).

I also expect that based on the literature review and results of the surveys there should be some prioritization of the recommendations and therefore indicators that could guide the development of a framework and also tools and guidelines.

**Author response:** Prioritisation of recommendations derived from the Phase I & II was undertaken in the third phase of framework development. The third phase, which utilised a Delphi survey involving key healthcare stakeholders, identified priority recommendations feasible for implementation. These specific recommendations guided framework development and informed the framework components (Results section, page 16-17, line 375-391).

Writers of this paper also suggest that NTDs are prone to outbreaks or epidemics which require epidemic or emergency preparedness and response, which is unusual since NTDs are generally known to be chronic conditions and have different data needs from other outbreak-prone conditions. Also, it was not discussed that PC-NTDs data collection are often one-off activities contrary to institutional data collection for conditions like malaria which involve routine data and facility-based data collection. PC-NTD data collection is also community-based with minimal and often insignificant facility-based data collection. These are important points that should have been discussed in the paper.

**Author response:** The statement on NTDs being classified as epidemic-prone diseases has been rectified. NTDs are categorised as diseases of public health importance and targeted for elimination or eradication (Background section, page 4, line 96-100). The need for the diseases to be easily notifiable within existing surveillance systems is to ensure prompt detection and response to limit disease re-emergence following elimination. The critical role played by the community towards improved community-based surveillance has been emphasised and discussed as suggested (Discussion section, page 49-50, line 727-734).

There also seems to be a mix in the presentation between data needs and operational issues and this needs to be clarified in the paper. The paper could also be better informed by a description of the health system for a better understanding of the M&E and surveillance needs at the different levels with a focus on the sub-national.

**Author response:** The framework components distinctively differentiate data needs (inputs: for improved reporting activities) and surveillance operational issues (processes: for improved overall surveillance activities) in the planned work phase (Fig 5, page 45, line 631). In addition, a description of the health system focusing on the surveillance system at the sub-national level has been provided (Methods section, page 9, line 218-223).

All authors reviewed the final version of the revised manuscript and ensured it adheres to all editorial requirements. We hope that our response to reviewers’ comments render the revised manuscript suitable for publication in *PLOS Neglected Tropical Diseases* journal.

Yours Sincerely,

Arthur Kipkemoi Saitabau Ng’etich

(Corresponding author)
